# American Hard Rubber Co. against John T. Toland

**Published:** 1863-07

**Authors:** 


### THE AM. HARD RUBBER CO. *vs.* JOHN T. TOLAND.

Frequent enquiries have been made in regard to a suite of the Rubber Co. against Toland for infringement of the Hard Rubber Patent. Upon making inquiry we find it to stand thus.

On the 7th of last April in the Circuit Court of the United

States, Southern District of Ohio, before Judges Swayne and Leavitt, of said Court, the following proceedings were had, to-wit:

HENRY B. GOODYEAR, ADM'R, ETC.,<br>*vs.*<br>JOHN T. TOLAND. } *In Chancery.*

And now comes the plaintiff by Mr. Stanberry, his counsel and this case is heard by the Court, here upon the bill answer, replication, depositions and exhibits, and thereupon the Court do find that the said two Letters Patent referred to in said bill, being Reissued Patents, Nos. 556 and 557, were duly granted and issued to said plaintiff, as administrator of said Nelson Goodyear, deceased, and that the same were and are in all respects valid and secure to said plaintiff, as such administrator the exclusive right of making, constructing, using and vending to others to be used, the said improvements specified in said letters patent, for the unexpired term of fourteen years, from the 6th of May, 1851.

And the Court doth further find that the said defendant hath been engaged in selling and manufacturing large quantities of plates and bases for artificial teeth and other articles made of the new substance, described and secured by said Letters Patent, in violation of said exclusive right so secured to the plaintiff as aforesaid, and without the license or authority of the plaintiff.

Whereupon it is by the Court here ordered that the said defendant be and he is hereby enjoined and forbidden from henceforth during the continuance of said Letters Patent, and during the term thereof yet unexpired, or any further term for which the same may be renewed or extended from continuing the manufacture and sale of plates and bases for artificial teeth, or other articles so made in violation of the said patents or either of them, and that the defendats pay the costs of the suit.

UNITED STATES OF AMERICA,<br>SOUTHERN DISTRICT OF OHIO. }

I, Joseph H. Geiger, Clerk of the Circuit Court of the United States for the 7th Circuit, and Southern District of Ohio, do hereby certify that the foregoing decree is truly taken and copied from the Journal of this Court.

[SEAL.] In testimony whereof I do hereunto subscribe my name and affix the seal of said Court, this 5th day of May, 1863.

JOSEPH H. GEIGER, *Clerk.*

Mr. Toland did not appear nor make any defense in the case from the fact, we suppose, that he was not in a position to do so; being absent, and his attention wholly diverted in other channels.

It will be observed that no damages were ordered, but simply an injunction granted, restraining him from the manufacture or sale of plates and bases for artificial teeth or other articles made in violation of the said patents during their unexpired term. As to whether an effort will be made to open up the case again, we do not know, neither are we advised as to whether it could be, were an effort made. T.

